# Contribution of oral narrative textual competence and spelling skills to written narrative textual competence in bilingual language-minority children and monolingual peers

**DOI:** 10.3389/fpsyg.2022.946142

**Published:** 2022-08-23

**Authors:** Giulia Vettori, Lucia Bigozzi, Oriana Incognito, Giuliana Pinto

**Affiliations:** Department of Education, Languages, Intercultures, Literatures and Psychology (Psychology Section), University of Florence, Florence, Italy

**Keywords:** bilingual language-minority children, textual competence, oral narrative, written narrative, spelling skills, Italian, Chinese

## Abstract

This study investigates the developmental pattern and relationships between oral narrative textual skills, spelling, and written narrative textual skills in monolingual and bilingual language-minority (BLM) children, L1-Chinese and L2-Italian. The aims were to investigate in monolingual and BLM children: (1) the developmental patterns of oral and writing skills across primary school years; (2) the pattern of relationships (direct and mediated) between oral narrative textual competence, spelling skills, and written narrative textual competence with age and socio-economic status (SES) taken under control. In total, 141 primary school children from grades 2 to 5 in Central Italy (44% BLM, 56% monolinguals) aged between 7 and 11 years (M-age = 8.59, SD = 1.13; 41% girls, 59% boys) obtained scores for oral and written narrative textual competence, spelling accuracy in dictation, and written texts. One-way ANOVA and ANOVA with robust method (Welch test) analyses and Bonferroni’s correction showed that BLM children had poorer spelling skills in dictation and written narrative textual competence (i.e., text structure) than their monolingual peers. After preliminary correlation analysis, the results of hierarchical regression showed that the relationship between oral and written narrative textual competence is completely mediated by spelling accuracy in BLM children. These results suggest that adequate performance in written narrative textual competence depends on adequate spelling accuracy in writing stories. The Sobel test verified the power of this mediation. In monolinguals, the strongest predictor of written narrative textual competence is oral narrative textual competence. This relation is stronger in older children whose spelling skills are automatized. The identified pattern of relationships shows a complex network of oral and written processes. The scarce spelling skills characterizing BLM children may explain why spelling skills determine a low written narrative textual level. Scarce spelling skills absorb cognitive resources, hindering high-level cognitive processes that regulate narrative production. In monolinguals, the medium of writing does not impact narrative textual competence. Children’s oral narrative textual competence easily transfers into their written narrative productions. These findings have implications for the assessment and instruction of literacy skills in young BLM children and their monolingual peers.

## Introduction

An exponential growing number of students in school classrooms worldwide are bilingual language-minority (BLM) children. These children experience the most intensive and sustained exposure to their second language (L2) when they begin formal education because they come from homes where the language background is different from the societal language (e.g., Hoff et al., 2021). Previous studies indicate that BLM children have fewer emergent orthographic knowledge in kindergarten (Incognito et al., 2021) and writing skills in school years (Goodrich et al., 2016) with detrimental effects on achievement and school outcomes. A serious disadvantage also for online communication in written form, a widespread practice (Alarcón et al., 2020). Theory and evidence indicate that writing narrative production skills are closely linked to the development of oral and spelling skills. Therefore, this study aimed to examine the developmental pattern and (direct and mediated) relations of L2 oral and writing skills among school-age children, including BLM children and their monolingual peers. Previous research shows the development of spelling and written narrative textual competence skills in monolingual, whereas oral narrative textual competence skills are less explored, especially across primary schools. Little is known about the developmental pattern and relations between oral and writing skills in BLM children acquiring two structurally and morphologically distinct languages. To our knowledge, this is one of the few attempts to study different skills across the primary school years in the profile of BLM children acquiring L1-Chinese and L2-Italian.

### Impact of oral language and spelling skills on writing skills

In this study, we refer to a model of writing development that comprehends important distal and proximal factors. Models of writing development, based on English, such as the “simple view of writing,” describe how proximal lower-level transcription skills (e.g., spelling skills), distal higher-level processes (e.g., planning, translating, and reviewing), and general cognitive skills (e.g., working memory, reading, attention) contribute to producing written texts. The updated model of writing, the so-called “not-so-simple view,” includes executive functions (e.g., attention, planning, reviewing) as core, describing how these components evolve and interact with transcription skills (handwriting or typing and spelling) in text generation. Consistent with previous models, mainly derived from opaque orthographies, beginning writers are mainly engaged in managing “lower-level processes,” such as proximal spelling skills (Hayes, 2012). Researchers agree that this step is particularly challenging for novice writers who are engaged in translating their ideas into text (Graham and Harris, 2000) by observing the grammatical and spelling rules of the language system (Graham, 2006). Recently, oral language and reading skills have been incorporated into these models as distal factors that contribute to the production of written text (e.g., Kim and Park, 2019). The recent “Direct and Indirect Effects model of Writing” (DIEW; Kim and Schatschneider, 2017; Kim, 2020) further demonstrates that both oral text generation and spelling skills are necessary to support writing quality. In novice writers, difficulties in spelling skills may hinder writing narrative textual processes and interfere with the transfer of oral narrative textual competence to written narrative textual productions. It is important to consider that the impact of spelling skills on writing texts may vary according to the characteristics of orthographies. Most evidence derived from studies typically focuses on English, where children’s spelling skills are challenged by an opaque language system with a strong inconsistency between its phoneme and grapheme correspondences. This research emphasizes the importance of investigating writing development in relation to children’s linguistic and socio-economic backgrounds (e.g., Incognito et al., 2021). We still know only very little about the role of spelling skills in writing texts in school-age BLM children acquiring Italian as L2. Italian is defined as a transparent or “shallow” language because it shows consistent sound-sign matching, similar to Finnish, German, and Spanish. Research indicates that for Italian monolingual children, the consistency between sound and sign matching sustains them in advancing quickly to writing sentences, and by the end of the first grade, children are able to write short texts (Bigozzi et al., 2016a). Further studies in Italian monolingual children (Bigozzi and Vettori, 2016) indicate that only in the case of a significant disadvantage in spelling skills do first graders’ oral narrative textual competence cease to sustain written narrative textual competence. Little is known about the pattern of (direct and mediated) relationships between oral narrative textual competence, spelling skills, and written narrative textual competence in children acquiring transparent Italian orthography through primary school years.

### Writing a story in L2

In studying children’s written narrative textual competence, it is interesting to distinguish which factors are sustained in the early or late phase of learning to write in primary school, because the characteristics of orthography may exert a different role on novice or more expert writers and monolingual or BLM children. Previous longitudinal studies on the transition from preschool to formalized education (e.g., Pinto et al., 2015, 2016) indicate that Italian preschool children’s oral narrative textual competence influences their later written narrative competence in first and second grades via a mediational effect of orthographic competence. Instead, there has been little research on how L2 spelling skills contribute to L2 written narrative textual competence in BLM children acquiring two orthographically distant languages: L1-Chinese (non-alphabet) and L2-Italian (alphabet and transparent orthography). The limited L2-Italian language input in the family context can negatively impact the acquisition of adequate L2 spelling skills (Roch and Hržica, 2020). Prior research indicates that BLM children use word knowledge in their L1 when writing in their second language (L2) (e.g., code-switching between two languages) (e.g., Gort, 2001). This adaptive strategy sometimes conducts to a “negative transfer” across languages (e.g., Howard et al., 2012), leading children to commit L1-influenced spelling errors when writing in the societal language (e.g., “l” instead of “r”). The Italian language as a transparent orthography might reduce cognitive demands on young writers in spelling, but less is known about the relationship between spelling and narrative texts when writers must manage the demand of the morphosyntactic complexity of this language.

## Aim and hypothesis

In this study, we examine the contribution of oral language and spelling skills to written narrative textual competence in a specific population of school-aged BLM children acquiring distant structural and morphological languages, L1-Chinese (non-alphabet) and L2-Italian (alphabet and transparent orthography), and their monolingual peers. Previous research has mainly focused on the transition from preschool to formalized education (e.g., Pinto et al., 2009, 2012; Bigozzi and Vettori, 2016). In this study, we focus on the important period of primary school years when children from grades 2 to 5 are expected to have almost automatized orthographic competence in Italian. Previous results have confirmed the usefulness of adopting both dictation and spontaneous spelling of words to assess children’s orthographic skills (Bigozzi et al., 2016a). In this study, two measures of spelling skills were adopted to gain a more complete picture of the involvement of spelling skills in the process of writing text by using both a measure *in the process* of writing text and an *external measure* of the level of proficiency in a dictation task.

This study contributes to closing the aforementioned gaps with the following aims.

First, we aimed to investigate the developmental patterns of BLM and monolingual children’s oral narrative textual competence, spelling skills, and written narrative textual competence across primary school years. We expect an improvement in children’s capacity to tell and write stories and in their spelling accuracy across primary school years. Specifically, we are interested in providing a better understanding of this improvement in the outcome measures of children in primary school by verifying floor or ceiling effects: whether there is rapid development or only gradual improvements during that period and whether there are any developmental plateaus.

Second, we aimed to examine the patterns of direct and mediated relationships between oral narrative textual competence, spelling skills, and written narrative textual competence in school-aged BLM children and their monolingual peers. We expect oral narrative textual competence and spelling skills to play a significant predictive role in written narrative textual competence. Specifically, we expect that the contribution of spelling skills is influenced by the level of proficiency, which is expected to be lower in novice writers and BLM children in comparison with monolinguals. Regarding monolingual children, we expect spelling skills to have a less significant influence on their written narrative textual competence, increasing with primary school grades, considering that most children show an adequate mastering of spelling skills in the Italian transparent orthography since the early years.

## Materials and methods

### Participants

A total of 141 primary school children aged 7–11 years living in Italy (M-age = 8.59, SD = 1.13; 83 girls and 58 boys) participated in this study as follows: 79 children (56%) were monolinguals, Italian-as-L1 children, exposed to the societal language both at home and school, and 62 children (44%) were Chinese-speaking language-minority bilingual children exposed to an L1 other than Italian in the family context. All Chinese children are born in Italy to parents who are both Chinese. In this study, the great proportion of Chinese as children’s minority language is related to the fact that they belong to a wide area in central Italy with a long history of Chinese immigrants. Given that, in Italy, children usually go to the public school closest to where they live, and classrooms saw a significant presence of Chinese bilingual language minorities. Children attending different primary school classrooms were included in this study: second grade *N* = 30 (monolingual = 14; BLM = 16); third grade *N* = 35 (monolingual = 20; BLM = 15); fourth grade *N* = 43 (monolingual = 25; BLM = 18); and fifth grade *N* = 33 (monolingual = 20; BLM = 13). Children with any known special educational needs or impairments/disorders were excluded to avoid any additional difficulties that could potentially affect their performance. School authorities, parents, and children provided consent to participate in the study. Background information about home language characteristics and sociocultural-economic status, defined as parents’ educational level (i.e., International Standard Classification of Education; ISCED-11; UNESCO Institute for Statistics, 2012), were collected using a parental questionnaire attached to the informed consent sheet. In Italy, first-grade teachers focus primarily on the spelling component of writing, whereas second-grade teachers focus on the textual properties of writing because second graders are expected to have finalized the acquisition of orthography (National Indications of the Italian Ministry of Education, hereafter MIUR, 2012). The children’s ages range from 7 to 10 years, corresponding to the second to fifth grades in school.

### Measures

#### Narrative tasks: Oral and written narrative textual competence

Two narrative tasks were adopted to measure children’s oral and written narrative textual competence. In a collective session in the classroom during school, the children individually performed a written narrative task. Each child wrote an invented story. Furthermore, in an individual session in a quiet room next to the classroom, children individually performed an oral narrative task. Each child was asked to tell a story they invented. Each story told was transcribed and codified. With respect to the aims of this study, each written and oral narrative was codified for the index of text structure. To identify the level of text structure in children’s written and oral stories, in line with previous research (e.g., Spinillo and Pinto, 1994; Pinto et al., 2020), the presence/absence of the 8 elements which characterize the narrative genre (e.g., title, opening, setting, description of character/s, problem, central event, resolution of the problem, and story ending) were identified to assign the corresponding score on a five-point scale as follows:

*Score 1 – no narrative*: Simple description or list of events, objects, or facts;

*Score 2 – sketch narrative*: Opening, setting, character(s), conclusion or opening, the sketch of the problem, and resolution;

*Score 3 – incomplete narrative*: Opening, character(s), problem, and resolution;

*Score 4 – essential narrative*: Opening, character(s), problem, central event, and resolution;

*Score 5 – complete narrative*: Title, opening, character(s), setting, problem, central event, resolution, and narrative closing.

The rate of agreement between the judges was 99%; cases of disagreement were resolved through discussion. In summary, each child in this study obtained a score for

• “oral narrative textual competence,” derived from the score of structure in oral texts;
• “written narrative textual competence,” derived from the score of structure in written texts.

#### A dictation task and a written narrative task: Spelling skills

To assess children’s spelling skills, a paper-and-pencil text dictation was performed individually by children in a collective session in the classroom during school time. The dictation task was taken (BVSCO) from the “Battery for the Evaluation of Writing and Orthographic Competence in Primary School” standardized for the Italian population. The children listened to a recorded text, and each child had to write down the text. To measure children’s spelling skills in the two tasks (dictation and written narrative), the orthographic errors were identified based on the classification of the orthographic errors by Pinto et al. (2012) which covers the entire variability of orthographic errors that children may commit in the Italian language. As indicated in previous research (see e.g., Bigozzi et al., 2017), it is important to gain a comprehensive score of orthographic error including both the cases in which the pronunciation of the target word is preserved despite the spelling violation (e.g., “anno” [year] instead of “hanno” [they have]), and both the cases in which the pronunciation of the target word is changed due to a spelling violation (“mecrato” instead of “mercato”). All phonemes are possible sources of non-homophone errors such that spelling errors of this type result in phonetically implausible words. Writing accuracy was determined by the total number of orthographic errors (see, Pinto et al., 2012), which were counted as many times as the error occurred.

#### Sociocultural-economic status

A parental questionnaire attached to the informed consent form was used to collect information about the children’s family sociocultural-economic status (SES). For this study, the index of ISCED level was considered as follows: a score from “ISCED 1 – Primary education” till “ISCED 6 – bachelor’s or master’s degree” was given for the educational level of fathers and mothers. Children’s SES scores were calculated based on the higher ISCED level between parents. The measure consists of the number of years of father and mother’s education (see also, Haman et al., 2017).

### Data analysis

First of all, we grouped the participants as follows: the youngest were the students attending second and third grades (total *N* = 65; monolingual = 34, BLM = 31); the oldest were the students attending fourth and fifth grades (total *N* = 76; monolingual = 45, BLM = 31). This division stems from the fact that second graders and third graders have similar spelling skills, in fact, it is at the end of the third grade that spelling proficiency is considered acquired. The first graders are not part of the sample because they are too immature in terms of learning. By third grade, they have acquired spelling skills, and in fourth and fifth-grades, children master spelling.

**FIGURE 1:**
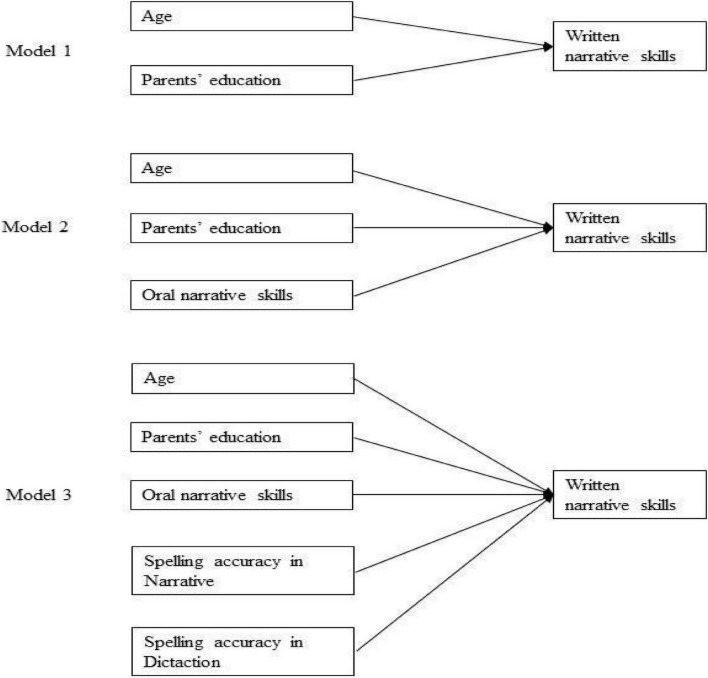
Hierarchical regression models.

Before analyzing the data, according to Tabachnick and Fidell’s (2013) recommendation, the presence of univariate outliers in the oral and written narrative skills scores was checked. No outliers are observed. Preliminarily, the homogeneity of variances was checked by Levene’s test. To investigate the oral narrative, spelling, and written narrative text production skills in L2-Italian shown by BLM (L1-Chinese and L2-Italian) and their monolingual Italian-speaking peers, one-way ANOVA was used. In the case of non-homogeneity of variances, ANOVA with robust methods was used (Welch’s Test). Moreover, we used Bonferroni’s correction to control the family-wise error rate (α_new_ = α_old_/n). Correlation analyses were then performed to investigate the relationships between variables as preliminary analyses for regression assumptions. Finally, we used hierarchical regression analysis to verify the increment in variation accounted for by the addition of predictors over a set of models (Figure 1). This is generally assessed by testing the change in R-square from one model to the next. If, after the inclusion of predictors at a given step, the R-square change is significantly greater than zero, we infer that the predictors added at that step offer incremental predictive power. The R-square change (increment) from Model 1 to Model 2 is computed as ΔR^2^ = Model 2 R^2^ – Model 1 R^2^. The R-square change (increment) from Model 2 to Model 3 is computed as ΔR^2^ = Model 3 R^2^ – Model 2 R^2^ (Darlington and Hayes, 2017).

To calculate the probability, if any, we used the Sobel test when the indirect effect of an independent variable on a dependent variable through a mediating variable is significant. Many studies rely on mediating models, and identifying whether a mediating variable significantly mediates the influence of an independent variable on a dependent variable is critical when assessing the value of such models.

## Results

Before analyzing the data, based on the parents’ educational level, the sample was distributed as follows: 1.4% primary school, 35.5% middle school, 25.5% 3-year professional qualification, 32.6% high school, 1.4% another higher education qualification other than a high-school diploma (conservatory, arts), and 3.5% master’s degree. Preliminary descriptive statistics and comparative analyses between monolinguals and BLM (one-way ANOVA and ANOVA with the robust method – Welch’s test) for the main variables are shown in Table 1. Using Bonferroni’s correction, the new alpha level is 0.01. The results show that statistically significant differences were found between monolingual and BLM children in spelling inaccuracy in dictation and written narrative skills. Specifically, BLM children exhibited a significantly higher number of errors than monolingual children. Regarding written narrative skills, BLM children performed statistically worse than their monolingual peers.

**TABLE 1:** Descriptive analyses and one-way ANOVA and ANOVA with robust methods results.

		Mean (SD)	Minimum	Maximum	Levene’s statistic^1^	F (Welch test)	DF
Spelling accuracy in Dictation	Monolingual	0.13 (0.15)	0	0.68	6.70*	11.51***	1,123
	BLM	0.22 (0.17)	0	0.65			
Spelling accuracy in Narrative	Monolingual	0.07 (0.06)	0	0.31	25.31***	5.60	1,123
	BLM	0.11 (0.14)	0	0.50			
Written narrative skills	Monolingual	3.10 (1.16)	1	5	0.42	21.61***	1,123
	BLM	2.14 (1.16)	1	4			
Oral narrative skills	Monolingual	3.17 (1.04)	1	5	0.98	4.54	1,98
	BLM	2.69 (1.10)	1	4			

*p < 0.05; ***p < 0.001; ^1^ in the case of significance of Levene’s test: F statistics were computed with Welch test; Bonferroni’s correction was applied, and the p-value is significant when p < 0.01.

Table 2 shows descriptive statistics and comparison analyses between younger and older children in monolinguals and BLM (one-way ANOVA and ANOVA with the robust method – Welch’s test). The results show that both in monolingual and BLM, performance in written narrative skills and orthographic accuracy improve with age. However, there is no significant increase in oral narrative skills. Using the Bonferroni Correction, the new alpha level is 0.01.

**TABLE 2:** Descriptive analyses and one-way ANOVA and ANOVA with robust methods results for monolingual and BLM children at school level.

		Mean (SD)	Minimum	Maximum	Levene’s statistic^1^	F (Welch test)	DF
**Monolingual children**							
Spelling accuracy in Dictation	Younger Children	0.22 (0.17)	0.03	0.68	13.99***	24.37***	1, 73
	Older Children	0.06 (0.08)	0	0.39			
Spelling accuracy in Narrative	Younger Children	0.10 (0.06)	0	0.23	1.66	10.43**	1, 65
	Older Children	0.05 (0.06)	0	0.31			
Written narrative skills	Younger Children	2.42 (1.06)	1	4	0.03	16.05***	1, 65
	Older Children	3.49 (1.03)	2	5			
Oral narrative skills	Younger Children	3.12 (1.17)	1	5	2.66	0.08	1, 64
	Older Children	3.20 (0.97)	2	5			
**BLM children**							
Spelling accuracy in Dictation	Younger Children	0.33 (0.15)	0.06	0.65	3.85*	34.68***	1, 55
	Older Children	0.12 (0.11)	0	0.46			
Spelling accuracy in Narrative	Younger Children	0.18 (0.16)	0	0.50	18.12***	16.46***	1, 56
	Older Children	0.05 (0.07)	0	0.32			
Written narrative skills	Younger Children	1.38 (0.77)	1	4	1.68	42.95***	1, 56
	Older Children	2.90 (0.98)	2	5			
Oral narrative skills	Younger Children	2.42 (1.24)	1	5	1.21	0.96	1, 33
	Older Children	2.83 (1.03)	2	5			

*p < 0.05; **p < 0.01; ***p < 0.001; ^1^ in the case of significance of Levene’s test: F statistics were computed with Welch test; Bonferroni’s correction was applied, and the p-value is significant when p < 0.01.

Correlation analyses were conducted to determine the relationships between the variables. Scores on written narrative skills were associated with the age of participants, oral narrative skills, and spelling accuracy in narration and dictation. Table 3 presents the results.

**TABLE 3:** Correlation analyses.

		Age	Parents’ education	Written narrative skills	Oral narrative skills	Spelling accuracy in Narrative	Spelling accuracy in Dictation
Monolingual	Age	–	−0.11	0.45**	0.04	−0.38**	−0.54**
	Parents’ education		–	0.01	0.04	0.14	0.07
	Written narrative skills			–	0.35**	−0.35**	−0.48**
	Oral narrative skills				–	−0.34**	−0.18
	Spelling accuracy in Narrative					–	0.76**
	Spelling accuracy in Dictation						–
BLM	Age	–	−0.21	0.66**	0.18	−0.48**	−0.62**
	Parents’ education		–	−0.25	−0.14	0.16	0.07
	Written narrative skills			–	0.35*	−0.51**	−0.63**
	Oral narrative skills				–	−0.37*	−0.23
	Spelling accuracy in Narrative					–	0.52**
	Spelling accuracy in Dictation						–

**p < 0.01; *p < 0.05.

Hierarchical regression analysis was performed to determine whether performance in oral narrative skills and spelling accuracy in narrative and dictation improved participants’ prediction of their written narrative skills beyond that provided by age and parents’ level of education. These predictors were used in the equation because of their statistically significant correlations with written narrative skills. Table 4 shows the standardized regression coefficients (β), R^2^, and change R^2^ (ΔR2) for monolingual children.

**TABLE 4:** Stepwise regression for monolingual children (dependent variable: written narrative skills).

		β	*R* ^2^	ΔR^2^
Step 1	Age	0.44**	0.19*	
	Parents’ education	0.06		
Step 2	Age	0.41**	0.32**	0.13
	Parents’ education	0.06		
	Oral narrative skills	0.36*		
Step 3	Age	0.30*	0.36**	0.04
	Parents’ education	0.07		
	Oral narrative skills	0.34*		
	Spelling accuracy in Narrative	−0.28		
	Spelling accuracy in Dictation	0.09		

*p < 0.05; **p < 0.01.

In Model 1, age and parents’ level of education accounted for significant variations in written narrative skills (R-square = 0.19, *F*(2,52) = 6.20, *p* = 0.004). In Model 2, age, parents’ level of education, and oral narrative skills accounted for significant variations in written narrative skills (R-square = 0.32, *F*(3,51) = 8.18, *p* < 0.001). The change in R-square from Model 1 to Model 2 was 0.13, reflecting a significant increase in the explained variation [*F*(1,51) = 9.99, *p* < 0.01]. In Model 3, the predictors accounted for significant variation in written narrative skills (R-square = 0.36, *F*(5,49) = 5.57, *p* < 0.001). The change in R-square from Model 2 to Model 3 was 0.04, which does not reflect a significant increase in explained variation [*F*(2,49) = 1.44, *p* = 0.247]. These results show that the strongest predictor of written narrative skills in monolingual children is oral narrative skills. Moreover, the age of participants as a control variable is presumably contributing to independent variance, as suggested by previous correlational analyses. This is explained by the fact that the beta value decreased and the *p*-value increased for age, in Model 3.

**FIGURE 2:**
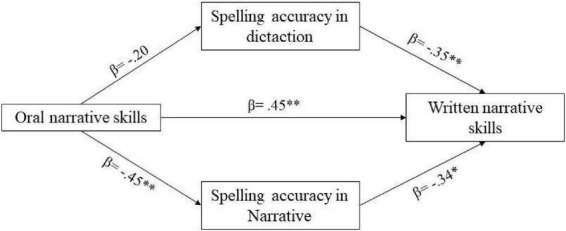
Mediational analysis model for BLM children. *p < 0.05; **p < 0.01.

Table 5 contains the standardized regression coefficients (β), R^2^, and change R^2^ (ΔR2) for BLM children. In Model 1, age and parents’ level of education accounted for significant variation in written narrative skills (R-square = 0.30, *F*(2,30) = 5.19, *p* = 0.004). In Model 2, age, parent’s level of education, and oral narrative skills accounted for significant variations in written narrative skills (R-square = 0.39, *F*(3,29) = 6.23, *p* = 0.002). The change in R-square from Model 1 to Model 2 was 0.09, reflecting a significant increase in explained variation [*F*(1,29) = 4.24, *p* < 0.05]. In Model 3, the predictors accounted for significant variations in written narrative skills, R-square = 0.54, *F*(5,27) = 3.71, *p* < 0.001. The change in R-square from Model 2 to Model 3 was 0.15, reflecting a significant increase in explained variation, *F*(2,27) = 4.39, *p* < 0.05. These results suggest that only spelling accuracy in written narrative texts is the best predictor of written narrative skills in BLM children; therefore, good performance in written narrative skills depends on good spelling accuracy in writing stories. Specifically, the model in step 3 improves significantly. In addition, in step 2, the oral narrative skills were significant, whereas, in step 3, they lost significance. This suggests that in the relationship between oral and written narrative skills, narrative orthographic accuracy completely mediates this relationship. We used the Sobel test to test the power of this mediation. The results indicate that there is a total mediation of narrative orthographic accuracy performance in the relationship between oral and written narrative skills (Sobel test statistic = 2.23, *p* < 0.05; Figure 2). Figure 2 explains the mediational model, and beta values and significances are shown for each distinct relationship.

**TABLE 5:** Stepwise regression for BLM children (dependent variable: written narrative skills).

		β	*R* ^2^	ΔR^2^
Step 1	Age	0.47*	0.30*	
	Parents’ education	−0.17		
Step 2	Age	0.39*	0.39*	0.09
	Parents’ education	−0.15		
	Oral narrative skills	0.31*		
Step 3	Age	−0.07	0.54**	0.15
	Parents’ education	−0.21		
	Oral narrative skills	0.23		
	Spelling accuracy in Narrative	−0.37**		
	Spelling accuracy in Dictation	−0.31		

*p < 0.05; **p < 0.01.

## Discussion

This study provides results on developmental patterns and relationships between oral narrative textual competence, spelling skills, and written narrative textual competence in two groups of school-age children, bilingual language-minority children, and their monolingual peers. The cross-sectional research design of this study provides results on the change and stability of the outcome measures throughout the primary school years.

As expected, oral narrative textual competence, spelling skills, and written narrative textual competence were found to develop throughout the primary school years in both language groups, BLM children and their monolingual peers, although some developmental trends were different within writing and oral domains and between language groups. From the comparison between young and older monolingual primary school children, a growth trend in oral narrative textual competence was observed. As presumably, older monolingual children produced oral stories with more structural information (i.e., story grammar units) than did the younger. This result shed light on a growth trend less documented in the Italian population and they are in line with previous studies on narrative development in diverse languages (Mäkinen et al., 2020). However, it is important to note that when considering BLM children’s oral narrative textual competence, the results show a developmental trend from the younger to older BLM primary school children with only minor improvement from the score around 2 (sketch narrative) to the score around 3 (incomplete narrative). Although a trend of improvement in oral narrative competence was observed both in bilingual and monolingual, the results did not reach significance, and this is somewhat surprising since narrative structure generally tends to improve significantly with age. The finding that the narrative structure in the BLM’s oral narratives reported only an improving trend, which was not statistically significant, is consistent with the fact that children’s oral narrative skills are poorly supported by teaching in primary school. Indeed, primary school focuses mainly on reading and writing skills, which, although related to the oral domain, are not yet able to influence the oral domain. In primary school years, children are scarcely engaged in telling oral narratives as for reading or listening to stories to the advantage of the teaching of writing (see e.g., Ministry of Education, University and Research MIUR, 2012).

Turning to spell skills, consistent growth in spelling accuracy occurred from younger to older primary school BLM and monolingual children. This is in line with previous studies on Italian children that document an age-related decline in errors, such as errors of omission, inversion, and non-phonological type together in dictation and written stories. This result can be confirmed for a specific population of BLM children acquiring the phoneme–grapheme correspondence, which is at the base of orthographic competence, in two languages with large differences. When taking a closer look at written narrative textual competence, performance was in the expected direction, with increasing primary school grades associated with improved performance in both language groups, BLM and their monolingual peers. Specifically, in monolingual children, the narrative structure in the younger group was around 2, indicating “sketch stories,” instead in the older group was a little above 4 moving toward an “essential narrative” with opening, character(s), problem, central event, and resolution. These results show increasing complexity in the mental model of the story, in line with previous studies (Pinto et al., 2015). In the younger BLM children group, the narrative structure was around 1, indicating a simple “description or list” of events, objects, or facts, whereas, in the older BLM children group, it was around 3, indicating an “incomplete story” with a conventional opening, character(s), problem, and resolution.

The two language groups (i.e., BLM and monolingual) display the same changes in oral and written domains across primary school years and are united by the school experience which focuses on reading and writing instruction while leaving aside oral practice. Alongside the similarities in growth, the comparison of oral narrative textual skills, spelling, and written narrative textual skills between monolinguals and BLMs shows that BLMs show quite equal oral narrative skills to monolinguals setting around the mean-level of 2 “sketch story” and 3 “incomplete story.” For what concerns the comparison of spelling skills, BLM children’s level of acquisition of sound-sign correspondence was significantly lower than those of their monolingual peers in the dictation task. As reported in the literature, a disadvantage at the orthographic level in BLM children has been observed since preschool when BLM children have reported lower levels of notational awareness than their monolingual peers (Incognito et al., 2021). Notational awareness refers to preschoolers’ conceptual knowledge of the writing system assessed by the “invented spelling task” requiring the ability to process forms of writing similar to conventional spelling. The invented spelling task, administered by the researcher, consists of asking the child to write a few words in order to assess, through various indicators, to what extent to which the child is able to produce signs more or less similar to letters and to read following with her/his finger what the child has written, in an attempt to make each sign correspond more or less correctly to a sound. Notational awareness is also assessed through the ability of the child to vary the number of signs in relation to the variation of the number of sounds in the word (Bigozzi et al., 2016a). Notational awareness represents a significant predictor of later reading and writing skills of primary school children in the Italian language system (Bigozzi et al., 2016b). In addition to the evidence that clarifies that children’s L2 exposure is strongly associated with their L2 language skills (e.g., Hoff, 2018), our results that BLM children were inferior in spelling skills in comparison to monolingual peers allow us to consider the influence of the characteristics of orthography, given that they were acquiring two distant structural and morphological languages, L1-Chinese (non-alphabet) and L2-Italian (alphabet and transparent orthography). While BLM children’s oral narratives are preserved, the obstacle of orthographic coding absorbs cognitive resources to the construction of a rich and detailed mental model of a story, aggravating the functioning of working memory for planning the text (Berninger and Swanson, 1994; Hayes, 2012). As previous writing models showed, children’s automatization of spelling skills enables them to free up memory and executive function resources to be devoted to higher-level processes of writing (e.g., generation of ideas, planning, and revision). This laborious search for words and correct transcription persists in older BLMs making the arduous circulation of knowledge between oral and written. These results of the comparisons between BLM vs. monolinguals corroborate the understanding of how important it is to master spelling so that it does not represent an obstacle to more creative and ideational processes such as textual writing, as already demonstrated in previous studies in other age groups (e.g., Pinto et al., 2015; Bigozzi and Vettori, 2016).

To successfully capture the pattern of the direct and mediated contributions of oral narrative textual competence and spelling skills to written narrative textual competence, we consider the results of BLM and monolingual primary school children. As predicted, spelling skills solely contributed to written narrative textual competence in BLM children, whose L2 spelling skills explained the most significant high proportion of the variance in written performance, even absorbing the influence of oral narrative textual skills in the statistical model. These results extend previous evidence about the relationship between oral language skills and sentence generation skills via spelling in the early stages of learning to write for English-speaking children to BLM children acquiring Italian as their L2. In our sample, the results show that the group of younger BLM children exhibit significant difficulties in L2 spelling skills in comparison to monolingual peers. Italian monolingual children, as highlighted in the literature, rapidly master spelling skills and begin to be more proficient in expressing their ideas and transferring their mental models into written texts. In this way, the medium of writing did not impact narrative textual competence, and oral narrative textual competence can easily transfer and influence textual writing production. It is important to consider monolinguals’ proficiency in spelling skills and related lexical knowledge in relation to their constant exposure to the societal language and the several text-hearing opportunities in the societal language since the early years. In addition, it is important to consider that, in primary school years, the teaching of writing is primarily focused on promoting the acquisition of orthographic accuracy in developing writers who are still facing lower-level transcription skills (spelling and handwriting) to progressively free working memory resources in favor of higher-level cognitive processes (Berninger et al., 1992; McCutchen, 1996; Graham and Harris, 2000) and writing strategies (planning, revising, and monitoring) (Arias-Gundín et al., 2021). Instead, BLM children show sustained L2 spelling difficulties. Such difficulties are a strong obstacle for writing texts in L2 to explain the larger proportion of variance and nullify the age effect. The BLM’s spelling difficulties in L2 negatively influenced their written narrative textual competence. When writing texts, children engage in high-level cognitive processes, such as planning, translating, and reviewing, while composing text (Berninger and Swanson, 1994; Hayes, 2012). The lack of automatization of L2 spelling skills in BLM children limits the cognitive resources devoted to high-level processes. Previous results (Vettori et al., 2022) on primary school BLM children show that BLM children fall significantly behind their monolingual peers in textual structure and lexical skills. This result can be linked to limited L2 vocabulary input in BLM children beyond school. It is interesting to consider the detrimental impact of low L2 spelling skills on school-age BLM children’s written narrative skills in relation to the different characteristics of Italian (L2 for BLM) and Chinese (L1 for BLM) languages. Italian is a transparent alphabetic language with complex morphology, whereas Chinese is a non-alphabetic language. BLM children are a heterogeneous group with regard to their L1 and L2 proficiency (Lonigan et al., 2018) and home literacy practices and experiences (Peets et al., 2022). As suggested by Wong (2017), children with L1 different from the societal language may show delayed development of their awareness of the adequate narrative structure qualities specific to the target language (Wong, 2017). Otherwise, they may not have the necessary linguistic resources to convey meaning appropriately (Kang, 2012).

In conclusion, our results confirm the importance of exploring direct and mediated relations between oral narrative textual, spelling skills, and written narrative textual competence (Pinto et al., 2015) and extend previous results focused on the transition between kindergarten to second grade by investigating primary school years. Our results underline the importance of considering how different writing systems pose different challenges for developing writers, especially when considering BLM children who receive limited L2 language input beyond school. Studying how oral and written narrative textual competence relate to each other via the mediating role of spelling skills will yield important implications for educational settings, where knowledge can be employed to design interventions that most effectively sustain L2 written narrative skills in BLM children speaking L1-Chinese at home. The results for monolingual school-age children highlight the need to support oral language, which impacts the quality of children’s narrative writing skills. It is also important to note that adequate mastering of spelling skills in the transparent Italian orthography no longer serves as a good discriminator of writing narrative skills in monolinguals.

### Limitations and future research

Our results form an important basis for future longitudinal research to gain a more comprehensive understanding of the relations between BLM and monolingual peers’ oral narrative, spelling, and written narrative skills. In a future study, a more fine-grained analysis accounting for the type of spelling and morphological errors would further inform the challenges posed by the characteristics of the Italian language to BLM children. In this study, the written narrative skills of children have been assessed using narrative text in L2. Future research can explore more in-depth BLM children’s narrative skills in both L1 and L2 to examine these relationships linked to reading and writing habits and motivation (Camacho et al., 2021; De Sixte et al., 2021). Future studies should examine a more heterogeneous language background of BLM children and compare the results from a cross-linguistic perspective. In addition, further research is required to verify the stability of the model detected in this study in BLM children with different language backgrounds.

## Data availability statement

The raw data supporting the conclusions of this article will be made available by the authors, without undue reservation.

## Ethics statement

The studies involving human participants were reviewed and approved by the University of Florence. Written informed consent to participate in this study was provided by the participants’ legal guardian/next of kin.

## Author contributions

All authors listed have made a substantial, direct, and intellectual contribution to the work, and approved it for publication.
