# Supplementary material for: Contribution of oral narrative textual competence and spelling skills to written narrative textual competence in bilingual language-minority children and monolingual peers
Source: Front Psychol. 2022 Aug 23;13:946142. doi: 10.3389/fpsyg.2022.946142 (PMC9445618; doi:10.3389/fpsyg.2022.946142)
Supplement: Supplementary file 1 [file Data_Sheet_1.pdf]

Supplemental material

Example of second graders' narrative text with scoring.

Once upon a time a little dog was looking on the ground and at a certain point it saw that there was a hole and inside there were many dogs. So the little dog went into the hole but then it couldn't get out, the mole heard it crying and asked it: "Why are you crying?" and the little dog said: "Because I can't get out, I want to get out", then the little dog managed to get out. [*Score 3 - incomplete narrative*: The story contains a conventional opening "*Once upon a time"*, the characters *"the dog" "the mole"*, the problem *"the dog couldn't get out from the dig"*, and resolution "*the little dog managed to get out",* but the story doesn't report the central event that explain how the dog managed to get out].
